# Genetics of canine subvalvular aortic stenosis (SAS)

**DOI:** 10.1186/s40575-021-00103-4

**Published:** 2021-05-07

**Authors:** Eric S. Ontiveros, Joshua A. Stern

**Affiliations:** Department of Medicine and Epidemiology, School of Veterinary Medicine, University of California Davis, Davis, CA 95616 USA

**Keywords:** Veterinary, Subaortic stenosis, Congenital heart disease, Aortic stenosis, Cardiac genetics

## Abstract

Subvalvular aortic stenosis (SAS) is one of the most common congenital heart defects of dogs. The disease is characterized by obstruction of the left ventricular outflow tract, resulting in pressure overload on the left ventricle. The etiology of obstruction is a fibromuscular nodule, ridge, or ring of tissue that increases aortic outflow tract velocity. This review is focused on the prevalence, inheritance pattern, and current genetic insights of canine SAS. The prevalence of this disease was reported at 4.7 % in a large veterinary referral hospital. The mode of inheritance for this disease has also been described in breeds with a high disease prevalence such as the Bullmastiff, Bouvier des Flandres, Dogue de Bordeaux, Golden Retriever, Newfoundland, and Rottweiler. Genetic investigations seeking to identify causative mutations for SAS are lacking with only a single published variant associated with SAS in Newfoundlands.

## Plain English Summary

Subvalvular aortic stenosis (SAS) is one of the most common heart conditions in large breed dogs that can result in fainting, a shortened life span, or sudden death. Boxers, Bouvier des Flandres, Bull Terriers, Bullmastiffs, German Shepherds, Dogues de Bordeaux, Golden Retrievers, Newfoundlands, and Rottweilers are diagnosed with SAS more often compared to other dog breeds suggesting a genetic etiology for this disease. Thus far, there has only been one reported genetic variant in the Newfoundland dog that is associated with SAS. Therefore, additional genetic studies remain warranted to deduce the genetics of this disease. In this review, we discuss the prevalence, inheritance pattern, and current genetics insights for canine SAS.

## Background

Subvalvular aortic stenosis (SAS) is a common congenital cardiac disease of dogs [1–3]. It is characterized by the presence of nodules, a ridge, or ring of fibrous tissue located below the aortic valve that obstructs the left ventricular outflow tract [4, 5]. Furthermore, this obstruction increases the pressure of the left ventricle resulting in increased aortic outflow tract velocity (AoV) [5, 6]. Mildly affected dogs tend to have a normal lifespan, but severely affected dogs have an average lifespan of 19 months [4]. Additionally, dogs that are severely affected by this disease have an increased risk of developing cardiac complications that include sudden death, congestive heart failure, syncope, and endocarditis [4]. The disease is challenging to manage as no treatment has been definitively proven to prolong the lifespan or quality of life for affected dogs. Surgical intervention such as resection of the stenotic tissue or balloon valvuloplasty have not resulted in a significant increase in the lifespan of affected dogs compared to pharmacologic intervention [7–12]. In a study by Sykes et al., there was a recurrence of clinical signs for this disease post-cutting balloon with combined high pressure balloon valvuloplasty procedure [13]. The Sykes et al. observation is further supported by Freedom et al., who notes that there is a recurrence of the subvalvular lesion post-resection [6]. Currently, moderate and severely affected dogs may be prescribed beta-blockers (atenolol), which has demonstrated a 56 month survival time in one study of severely affected dogs [9]. Although the results of the Meurs et al. [9] study are contested by an Eason et al. [14] retrospective study, which failed to note an increase in lifespan for severely affected dogs treated with atenolol, current clinical practice continues to prefer to use pharmacological intervention over surgical intervention for SAS affected dogs. All of these findings suggest that a genetic understanding of the disease will be crucial to our understanding of the molecular phenotype and possible novel drug targets. Additionally, genetic variants associated with SAS may provide genetic testing strategies to reduce disease prevalence in highly affected breeds.

The genetics of SAS continue to remain elusive in dogs affected by this disease. SAS is reported as one of the most common congenital heart defects in purebred dogs [3, 15, 16]. A similar type of lesion is also identified in humans affected by SAS, which consists of a ring located below the aortic valve restricting AoV [6, 17–20]. Furthermore, in humans, SAS is commonly seen in patients with Shone’s complex which consist of four obstructive lesions: coarctatation of the aorta, a parachute mitral valve, a supravalvular aortic ring, and a subvalvular aortic ring [6, 21]. The genetic etiology of SAS in humans remains mostly unknown. A previous report identified a 1.5–1.6 Mb deletion in a patient diagnosed with 3q29 microdeletion syndrome that had complications as a result of SAS, PDA, pulmonary hypertension, and meconium aspiration [22]. The deletion appeared to be inherited, as the patient’s father also carried the deletion. Similarly, not much is known about the genetic etiology of SAS in dogs. So far, there is only one genetic variant reported to be associated with SAS in North American Newfoundland dogs [23]. The variant is a one-codon insertion in the Phosphatidylinositol Binding Clathrin Assembly Protein (*PICALM)* gene identified in Newfoundlands [23]. The *PICALM* gene is an clathrin endocytosis mediator which is a process that occurs during embryological development of the heart [24]. In a follow-up study of European Newfoundland dogs, Drogemuller et al. did not replicate the previously reported *PICALM* association with SAS [23, 25]. This highlights the need for additional genetics studies in the Newfoundland dog as well as other dog breeds with a high prevalence of this disease. Given the similar pathophysiology of SAS in dogs and humans, dogs represent an excellent translational model for naturally occurring SAS [6]. Therefore, elucidating the genetic architecture of SAS in dogs can help reduce the prevalence of this disease in affected breeds as well as help decipher potential causes of this disease in humans. This review is focused on the phenotypic characterization, prevalence, inheritance pattern, and current genetic insights for dogs affected with SAS.

### Phenotypic characterization of SAS

A critical component of any genetic study is robust phenotyping. Phenotyping challenges may underscore one of the reasons that SAS genetic studies are so complex. Pyle and Patterson provided a road map to understanding the gross pathological characteristics of SAS in their seminal work on this condition in 1976 [5]. Here, they showed a wide degree of affected phenotypes, ranging from more subtle fibrous nodules to severe tunnel-like lesions of the left ventricular outflow tract. In general, the severity of disease is correlated to the severity of the heart murmur ausculted [23, 26–28]. In the Newfoundland breeding study, the severity of disease was not predicted by the breeding pair, meaning that even mildly affected individuals can produce severely affected offspring [5]. Furthermore, Pyle, et al. detailed the challenges in identifying mild or equivocal SAS by auscultation, identifying dogs with obvious subvalvular ridges in the absence of a heart murmur [29]. Pyle, et al. further described the progressive nature of this disease and noted that dogs need to reach skeletal maturity to fully comprehend the severity of this disease. Additionally, animals that were echocardiographically normal and had necropsy evidence of SAS were described, suggesting that, at the time of these investigations, echocardiography may have been insensitive for the phenotyping of this condition [29]. Since then, echocardiographic equipment and image quality have markedly improved, making it unclear whether phenotyping at necropsy is necessary for genetic studies.

**Fig. 1:**
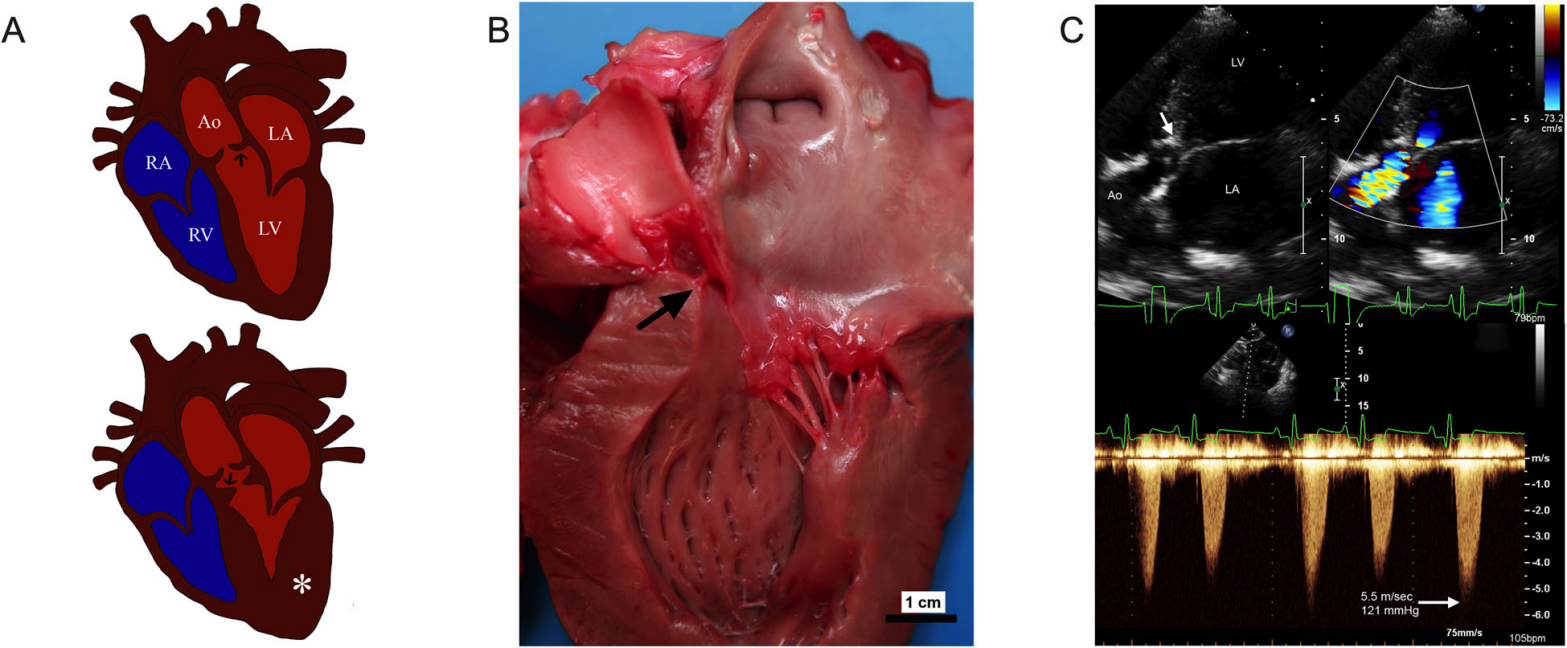
Depiction of the subvalvular lesion through a schematic drawing, pathology image, and echocardiography. **a** An illustration of a healthy (top) and SAS affected (bottom) canine heart. The arrow points to the aortic valve (top) and subvalvular ridge (bottom). This image was reprinted (with permission) from the Ontiveros et al. manuscript [33]. **b** A gross pathology image of a heart affected with SAS with the subvalvular ridge/ring denoted by the arrow. **c** 2D, color (upper image) and spectral (lower image) Doppler echocardiogram images of a dog with severe SAS. Note the turbulent blood flow and high aortic flow velocity consistent with severe subvalvular aortic stenosis. The subvalvular lesion is denoted by the white arrow in the top left panel of this image. The left ventricle (LV), aorta (Ao) and left atrium (LA) are noted

Echocardiographic assessment of SAS includes two-dimensional evaluation of the left ventricular outflow tract and critical evaluation for the presence or absence of any subvalvular lesions [Fig. 1] [30, 31]. In the absence of a two-dimensional lesion, the severity of SAS is typically assessed by measurement of the aortic outflow tract velocity. It has been demonstrated that variation in the placement and alignment of the Doppler echocardiographic transducer can impact this numerical assessment, leading to the recommendation that flow velocity be performed from the subcostal position by continuous-wave Doppler in an effort to reduce false negative results [30, 32]. The use of aortic flow velocity diagnosis, however, leaves room for a relatively wide equivocal zone of affection. Veterinary cardiologists have not uniformly agreed on normal versus abnormal cut-offs values for AoV. In the authors’ research and clinical practice, dogs with an aortic flow velocity < 2.0 m/sec are generally considered normal and over 2.5 m/sec are considered mildly SAS affected. This schematic leaves many dogs with values between 2.0 and 2.5 m/s at an uncertain disease status and at risk of being misphenotyped in genetic investigations. Therefore, it is reasonable for investigators to strive to use the phenotypic extremes, i.e. more severely affected compared to more clearly normal dog samples, thus avoiding the equivocal zone by a large margin.

Given all of this information, it is clear that the gold standard for genetic investigation should include animals that were evaluated by a board-certified veterinary cardiologist and phenotyped at minimum by high-quality 2D and Doppler echocardiography. We also want to note that breed specific AoV reference ranges for SAS affected dogs are warranted. Specific reference ranges will take into account the varying aorta size different dog breeds have due to their breed size. Furthermore, at the time of the echocardiogram, we want to ensure that the dog is not stress since stress can inadvertently elevate the AoV causing a dog that has a normal AoV to get categorized as equivocal. From the authors experience, even if stressed, a truly unaffected dog AoV will not reach the threshold to categorize it as affected (AoV > 2.5 m/s). Where possible, using samples that were confirmed at necropsy would be of great utility to provide confidence in disease categorization. From a practical perspective, investigators should avoid using auscultation as a phenotyping tool in SAS. Given the change in severity observed over time, investigators should also strive to use samples from individuals that are at least 9–12 months of age at the time of evaluation.

### Prevalence of SAS

It has been reported that certain dog breeds are overrepresented in the diagnosis of SAS. Dogs with a reported predisposition include Boxers, Bouvier des Flandres, Bull Terriers, Bullmastiffs, German Shepherds, Dogues de Bordeaux, Golden Retrievers, Newfoundlands, and Rottweilers [1, 2, 33–35]. Based on the increased incidence of SAS in these breeds, an underlying genetic cause has been implicated. The most commonly mentioned breeds that have a high incidence for SAS consists of the Boxers, Newfoundland, Rottweiler, German Shepherd and the Golden Retriever (Table 1). Of these breeds, the Golden Retriever and Newfoundland have been the most studied and illustrate that SAS is a heritable condition [5, 26]. Further studies remain warranted in other breeds with a high predisposition for SAS some of which will be discussed in this review. The prevalence of SAS in a large referral hospital was estimated to be 4.7 % for the cardiology service [33]. In our recent study, Bullmastiffs had the highest prevalence for this disease at 6.59 %, followed by Newfoundland at 4.46 % [33]. In a separate population of dogs, it was reported that SAS was the third most common congenital heart defect with the Dogue De Bordeaux breed having the greatest incidence of this disease and identified in 72.7 % of this breed seen at that hospital [3]. The Brambilla, et al. study further noted that SAS had a significant sex predilection, with males more frequently affected, and associated with patent ductus arteriosus [3]. All of these studies help highlight that SAS is most commonly seen in large and medium purebred dogs with few small or toy breed dogs reported.

**Table 1:** The breeds with the highest reported incidence for SAS in selected publications

Author	Study	Publication Year	Top SAS Affected Breeds	No. of SAS Dogs
Patterson [1]	Epidemiological and Genetic Studies of Congenital Heart Disease in the Dog	1968	• German Shepherd • Boxer • Newfoundland	40
Oliveira et al. [2]	Retrospective Review of Congenital Heart Disease in 976 Dogs	2011	• Boxer • German Shepherd • Dogue de Bordeux • Newfoundland • Rottweiler • Golden Retriever • Mongrel • Labroador Retriever	241
Ontiveros et al. [33]	Congenital Cardiac outflow Tract Abnormalities in dogs: Prevalence and Pattern of Inheritance from 2008 to 2017	2019	• Bullmastiff • Newfoundland • Boxer • Golden Retriever • Rottweiler • German Shepherd • Pitbull Terrier • Labrador Retriever • Mixed breed	259
Brambilla et al. [3]	Epidemiological study of congenital heart diseases in dogs: Prevalence, popularity, and volatility throughout twenty years of clinical practice	2020	• Boxer • German Shepherd • English Bulldog • Newfoundland • Rottweiler • Golden Retriever • Labrador Retriever • Dachshund • Dogue de Bordeux • Bull Terrier • Crossbreeds	296

### Mode of inheritance of SAS

**Fig. 2:**
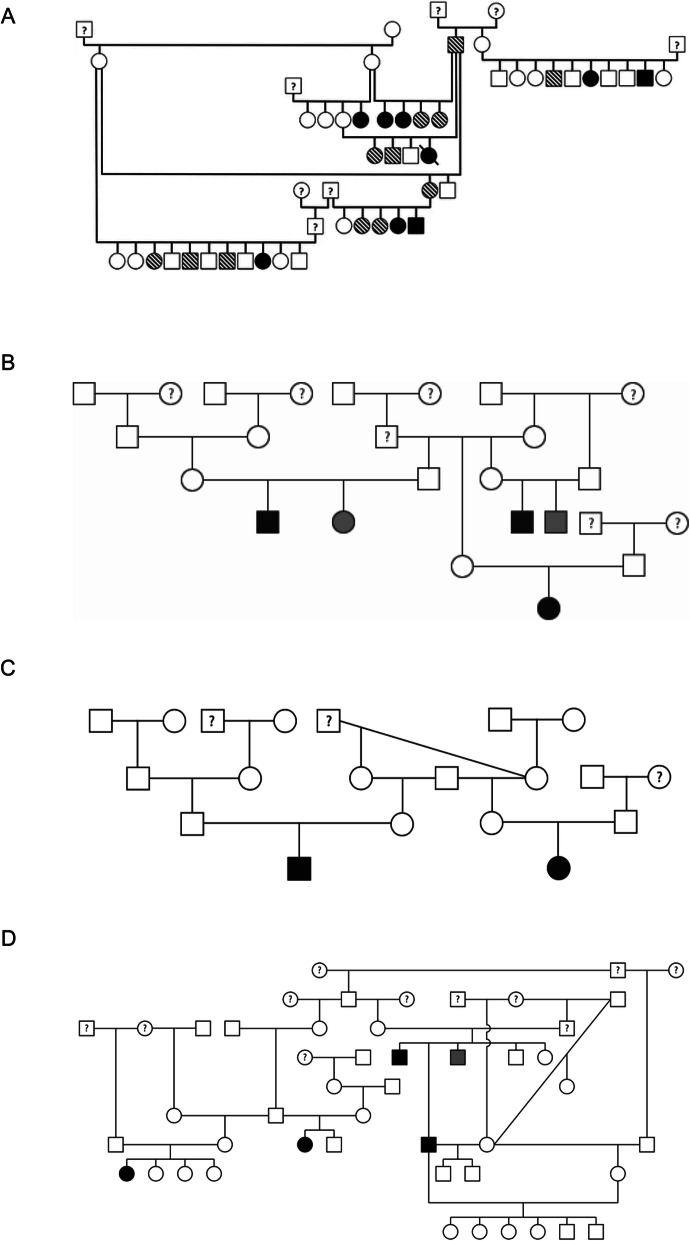
Pedigree representing a family of SAS affected (**a**) Newfoundlands, (**b**) Bullmastiffs, (**c**) Golden Retrievers, and (**d**) Rottweilers. These pedigree figures were originally reported in Stern, et al. and Ontiveros, et al. manuscripts [23, 33]. In the pedigree, square represent males, circles represent females, white squares or circles represent unaffected dogs, black squares or circles denote affected dogs, gray squares or circles denote equivocal dogs, striped square or circles also represent equivocal dogs, and square or circles with a question mark denote missing phenotypic information for those dogs

Pyle and Patterson’s landmark manuscript described the pathology of SAS based on breeding studies conducted in the Newfoundland dog [5]. This study conducted five different breeding crosses that included the following: (1) affected x affected cross, (2) affected x normal cross, (3) affected x normal non-Newfoundland (F_1_ generation), (4) F_1_ affected x affected Newfoundland (F_1_ backcross), and (5) F_1_ affected x normal non-Newfoundland [5]. All of these crosses produced 20 % or greater SAS affection status in the offspring, except for the F_1_ affected x normal non-Newfoundland cross which did not produce any affected dogs. Based on this breeding study, it is evident that breeding an SAS-affected dog results in transmission of this disease. Furthermore, this study also noted that SAS is a progressive disease, and that disease severity increases as the dog reaches maturity. This is the only report that utilizes prospective dog breeding to deduce the mode of inheritance for SAS affected dogs. Based on the affection status of the offspring in the mating crosses, a polygenic mode of inheritance or dominant mode of inheritance was suspected. The mode of inheritance for SAS affected Newfoundlands was also evaluated in the Reist-Marti, et al. manuscript which determine that it followed an autosomal co-dominant mode of inheritance [36]. In this study, pedigree data was evaluated for 230,000 Newfoundland dogs that traced back to the 19th century. Unfortunately, this study used unconventional, lower echocardiographic AoV thresholds that can result in normal unaffected dogs or equivocal dogs being classified as affected. Based on our experience and published normal values in dogs, those that have an AoV of 1.8-2 m/s are unaffected and not mildly affected. Furthermore, classifying dogs that have an AoV of 2 m/s or higher as severely affected results in artificial inflation of the disease incidence and severity. In another study that evaluated the mode of inheritance for SAS affected Newfoundlands via pedigree analysis, a more conventional echocardiographic AoV threshold was utilized. In this study by our research group, affected dogs were required to have an AoV ≥ 2.5 m/s and unaffected dogs had an AoV ≤ 1.9 m/s [23]. The pedigree consisted of 45 Newfoundland dogs, and the results support either an autosomal dominant mode of inheritance or polygenic inheritance [Fig. 2a]. It is crucial that future studies select AoV thresholds that confidently distinguish unaffected and affected dogs. For instance, studies that use lower AoV thresholds are more likely to result in false positive cases whereas studies that utilize higher thresholds might inadvertently miss mildly affected dogs. For a disease such as SAS where mildly affected dogs can produce severely affected offspring, it is crucial that genetic studies ensure that the threshold for AoV is critically assessed.

Previously published studies have demonstrated that the mode of inheritance for SAS in dogs is complex and variable [5, 6]. This is similar to humans, in which an autosomal recessive as well as an autosomal dominant mode of inheritance was reported previously [18–20]. In dogs, pedigree evaluation was also completed for other dog breeds, with a high prevalence for this disease [33]. An autosomal recessive mode of inheritance was reported for Bullmastiff, Golden Retriever, and Rottweiler breed (Table 2; Fig. 2b-d) [33, 35]. These pedigree reports demonstrate affected offspring being produced by unaffected parents or affected parents producing unaffected offspring. Pedigree evaluation for the Dogue de Bordeux breed also implicated an autosomal recessive pattern of inheritance [35]. The varying descriptions for the mode of inheritance within and among different affected breeds might be due to the possibility of multiple genetic variants causing this disease in subpopulations of a dog breed. This is supported by the findings of Pyle and Patterson where they suggested a polygenic mode of inheritance for SAS affected Newfoundlands [5]. Furthermore, this phenomenon has also been noted in other canine cardiac disease such as the Doberman pinscher affected with dilated cardiomyopathy [37, 38].

**Table 2:** Select publications detailing proposed mode of inheritance for SAS across various dog breeds

Author	Study	Year	Breed(s)	Mode of Inheritance
Pyle et al. [5]	The genetics and pathology of discrete subaortic stenosis in the Newfoundland Dog	1976	Newfoundland	Polygenic or autosomal dominant
Stern et al. [26]	Familial subvalvular aortic stenosis in golden retrievers: inheritance and echocardiographic findings	2012	Golden Retriever	Inconclusive, suggests autosomal recessive or polygenic inheritance
Resit-Marti et al. [36]	Genetic evidence of subaortic stenosis in the Newfoundland dog	2012	Newfoundland	Autosomal codominant with lethal homozygosity
Ohad et al. [35]	The occurrence and suspected mode of inheritance of congenital subaortic stenosis and tricuspid valve dysplasia in Dogue de Bordeaux dogs	2013	Dogue de Bordeux	Autosomal Recessive
Stern et al. [23]	A single codon insertion in PICALM is associated with development of familial subvalvular aortic stenosis in Newfoundland dogs	2014	Newfoundland	Autosomal Dominant (with incomplete penetrance) or polygenic
Ontiveros et al. [33]	Congenital Cardiac outflow Tract Abnormalities in dogs: Prevalence and Pattern of Inheritance from 2008 to 2017	2019	• Bullmastiff • Golden Retriever • Rottweiler	Autosomal Recessive

Based on the reported inheritance patterns in dogs affected by SAS, reducing the prevalence of this disease in most affected dog breeds will be challenging without the development of a genetic test. For instance, dog breeds that followed a recessive mode of inheritance will continue to produce heterozygous carriers that will go undetected since they will not show clinical signs of the disease. Therefore, these undetected dogs can remain in the breeding pool, further propagating this disease. For dogs that follow a dominant mode of inheritance, only one minor allele is required to show clinical signs of this disease. Thus, dominant inheritance would potentially make selection more effective at reducing disease prevalence, however the previous reports of incomplete penetrance present additional challenges to overcome in the absence of a known genetic mutation.

### Genetic studies of SAS

To date, a single variant has been reported in association with SAS. This is a single codon insertion in the *PICALM* gene identified in a family of North American Newfoundland dogs with SAS [23]. In this study, the genome-wide association study (GWAS) analysis failed to identify a locus associated with SAS in relatively small cohort of 18 cases and 20 controls. No locus of genome-wide significance was identified, suggestive regions were identified one of which harbored the identified *PICALM* variant [23]. This GWAS analysis results were influenced by population stratification based on the reported genome inflation factor of 1.87. The *PICALM* variant in Newfoundlands was identified via RNA sequencing (RNA-seq). The study utilized tissue from the subvalvular region and ridge from two affected Newfoundland dogs and from the subvalvular region in six control dogs from different breeds [23]. Coding variants located in the suggestive regions of interest in the GWAS analysis were evaluated and analyzed if they were present in both SAS affected Newfoundland dogs [23]. This led to the identification of a three-nucleotide insertion in *PICALM*, which adds a leucine to the amino acid sequence of this gene [23]. The prevalence of this variant was calculated to be 80.6 % in the broader Newfoundland population of that study and the manuscript demonstrated that this variant was not the sole causal variant of SAS within the breed [23]. This highlights the proposed polygenic inheritance for SAS, given that this variant did not explain all cases of SAS affected Newfoundland dogs [5, 6]. Another possibility is that this variant is not truly associated with SAS in Newfoundland dogs. In human genetic studies, both a discovery and a validation cohort are required to confirm a variant association with a disease. Although a replication study was attempted in a separate cohort of European Newfoundland dogs, the phenotype criteria used in this study differed from the Stern et. al publication by raising the unaffected AoV threshold to < 1.9m/sec and reducing the SAS affected threshold to > 2.4m/sec when echocardiographic data was available [23, 25]. Additionally, this replication study included some dogs categorized as unaffected by auscultation alone and a large number of dogs with unknown phenotype for which genotype-phenotype correlation was not possible [25]. Therefore, it remains possible that discrepancies in phenotyping may contribute at least partially to the differences found in this replication study [25]. The authors of this review remain confident that to truly ensure a dog is not affected by SAS, especially in a breed with a high disease prevalence for this disease, an echocardiogram is required to evaluate heart morphology as well as the AoV. The authors acknowledge that additional studies remain warranted to determine if the Newfoundland *PICALM* variant is truly causative for SAS or simply associated in the North American study cohort. A further possibility that could have resulted in differences between these two studies is that multiple variants might be responsible for causing SAS in the Newfoundland. Therefore, different subpopulations, especially if from different geographical locations, could have different variants resulting in their respective SAS phenotypes.”

A GWAS analysis was also completed for SAS affected Golden Retrievers [39]. This abstract reported a region on chromosome 13 that was identified in 29 affected and 52 control Golden Retrievers. We have also completed a GWAS analysis for the Newfoundland and Rottweiler breed, both of which also demonstrated a locus on chromosome 13 that exceeded the Bonferroni threshold for significance (unpublished data). Therefore, we completed an across breed GWAS analysis for the Golden Retriever, Newfoundland, and Rottweiler breeds to determine if they shared a common genetic variant on chromosome 13 (unpublished data). Given that these breeds share haplotypes with each other, it is possible that they may all share a genetic variant that predisposes them for SAS [40]. The across breed GWAS analysis identified a locus achieving genome-wide significance on chromosome 13. The aim of these analyses was to decipher the genetic architecture for SAS affected dogs. This candidate region on chromosome 13 requires further evaluation through the use of targeted or whole-genome sequencing approaches. This can result in the identification of distinct or shared genetic variants associated with SAS in the Golden Retriever, Newfoundland, and Rottweiler breed.

As evident, genetic investigations of SAS are limited and focused on dogs with a high prevalence for this disease [33]. Our currently unpublished data implicates chromosome 13 across three breeds that have been studied via GWAS analysis [39]. Furthermore, given the complexity of SAS, it is a possibility that rare variants with a large effect or multiple modifying variants can be responsible for the pathophysiology of SAS. This can make identifying variants associated with SAS complicated and should be considered in future studies. Novel techniques for identifying common molecular features of this disease across breeds may yield new drug targets and improve our understanding of this complex condition. This can include using RNA-sequencing in which tissue from the subvalvular ridge can be used to identify what genes are expressed in this area and if they are dysregulated. Furthermore, gene knockout analysis using *in-vitro* models such as zebrafish or mice can also be utilized to determine the effect candidate genes located in the region of association may have on cardiac development.

## Conclusions

Our understanding of the genetics of SAS in dogs has continued to improve since the landmark Pyle and Patterson breeding study of Newfoundland dogs [5]. However, genetic studies alone are not enough and must be complimented by epidemiologic and longitudinal clinical studies. A robust understanding of disease epidemiology is required as our understanding of true disease prevalence may be influenced by the fact that most SAS studies are performed at tertiary referral centers. Furthermore, the lack of successful interventional procedures may result in only a fraction of SAS cases being referred for tertiary care and subsequently result in an underestimated disease prevalence. Additionally, mildly affected dogs may go undetected due to a lack of clinical signs and allow continued disease propagation within the population. True longitudinal studies across a variety of disease severities are also lacking for SAS. All of this epidemiologic and clinical outcomes data would be of great benefit to compliment the ongoing genetic investigations.

Although there has been an increased effort and studies that aim to identify genetic variants associated with SAS, thus far only one genetic variant has been reported to be associated with SAS in dogs [23]. We have completed GWAS analyses for the Golden Retriever, Newfoundland, and Rottweiler breed. The results from these unpublished analyses all implicated chromosome 13 in individual and across breed GWAS. This is rather interesting as these three dog breeds do not necessarily descend from the same ancestor and have been bred for different functions based on genealogical assessment, although they do share haplotypes [40]. Therefore, it will be intriguing if all three breeds share a genetic variant that causes SAS. Another possibility could be that multiple variants affecting either the same or different pathways could be the cause of canine SAS. We do not yet know if the variants of interest will be common or rare with small or large effects on disease pathogenesis. Given the progressive nature of SAS in dogs, it is clear that this disease is complex and potentially polygenic. Therefore, as next-generation sequencing (NGS) costs continue to decline, this will provide an opportunity to further interrogate the genetics of SAS in breeds that have a high prevalence of this disease. This can be completed via whole-genome sequencing, long-range whole genome sequencing, RNA-sequencing, or perhaps most powerfully as a combination of multiple methods.

 Furthermore, functional analyses are warranted and should be pursued to stringently validate any associated variants as potential mutations. Immunohistochemistry can be utilized to first determine if the gene of interest is expressed in the subvalvular lesion for SAS affected dogs. This can ideally be followed by CRISPR gene editing to mimic the variants of interest and determine the true effect on cardiac development in model organisms. After variants are identified to be stringently associated with SAS, breeder education is essential to ensure that the prevalence of SAS can be reduced in affected dog breeds. It is also vital to educate breeders on how to utilize genetic testing in their programs to ensure a reduction in SAS affected offspring while preserving genetic diversity where possible.

Although the genetics of SAS continues to mystify investigators, the progress that has been completed thus far aids in unraveling the genetic architecture of this complex disease. As evident in this review, additional research regarding the genetics of SAS remains warranted to identify variants associated with this disease. We are hopeful with continued research and advancing science that identification of novel SAS associated variants can lead to a combination of reduced disease prevalence and improved understanding of disease pathways that may represent future novel therapeutic opportunities.

## Data Availability

Thedata discussed in this review article is published and cited.

## Abbreviations

SAS: Subvalvular aortic stenosis.
AoV: Aortic outflow velocity.
*PICALM*: Phosphatidylinositol Binding Clathrin Assembly Protein.
GWAS: Genome-wide association study.
